# Managing Blood Pressure Beyond Viral Suppression for Patient and Provider Perspectives on Antihypertensive Medication Adherence in HIV Care: A Qualitative Study

**DOI:** 10.21203/rs.3.rs-9476869/v1

**Published:** 2026-05-18

**Authors:** Victoria Ayodele, Joshua Kwawuvi, Mersedes Brown, Nwora Okeke, Juan Gonzalez, John Bartlett, Charles Muiruri

**Affiliations:** Duke University; Duke University; Duke University School of Medicine; Duke University School of Medicine; Duke University School of Medicine; Duke University; Duke University

**Keywords:** People with HIV, HIV providers, Antihypertensive, Medication Adherence, COM-B Framework

## Abstract

**Background:**

Hypertension is a leading contributor to cardiovascular disease morbidity among people with HIV (PWH), yet adherence to antihypertensive medications remains suboptimal. While HIV care has achieved high levels of medication adherence through structured monitoring and communication, less is known about how PWH and HIV providers perceive barriers and possible interventions to antihypertensive adherence within HIV care settings.

**Methods:**

Semi-structured interviews were conducted with 20 virally suppressed PWH diagnosed with hypertension and 19 HIV providers between August 2022 and February 2024. Interviews explored experiences and perceptions related to antihypertensive medication adherence. Data were analyzed using a rapid qualitative approach with matrix-based techniques and mapped to the Capability, Opportunity, and Motivation (COM-B) behavior change framework.

**Results:**

PWH and HIV providers identified overlapping barriers to antihypertensive adherence spanning all COM-B domains. Shared motivational barriers included concerns about side effects, pill burden, and low perceived urgency of hypertension. Opportunity-related barriers included competing life demands and limited visibility of nonadherence due to blood pressure variability. Capability-related challenges centered on difficulty communicating and understanding long-term hypertension risk compared with HIV. Divergence of perspectives emerged around communication, with PWH frequently describing misunderstandings about instructions and intentional non-disclosure of nonadherence, whereas HIV providers emphasized limited capacity to manage non-HIV conditions.

**Conclusions:**

Antihypertensive medication nonadherence among PWH reflects patient-, provider-, and system-level challenges that are not fully addressed within HIV care. Extending the strengths of HIV care through structured communication, monitoring, and care delivery offers a practical pathway to improving cardiovascular outcomes among PWH.

## BACKGROUND

Hypertension is a leading cause of cardiovascular disease (CVD) among people with HIV (PWH).(1, 2) It is estimated that globally 35% of PWH have hypertension and PWH with hypertension have a two-fold heightened risk of incident acute myocardial infarction compared to PWH without hypertension.(3) Antihypertensive medication nonadherence is a leading cause of inadequate blood pressure control in this population which is associated with poor CVD outcomes.(4)

HIV viral suppression is largely driven by optimal antiretroviral therapy adherence (ART) and, consequently, medication adherence research for PWH has largely focused on ART adherence with limited focus on non-AIDS comorbidities. Studies have compared ART and comorbidity medication adherence among PWH have produced mixed results.(5, 6) However, there have been others to report low adherence to comorbidity medication, as patients perceive ART more sufficient than hypertension or other chronic treatments.(3, 7) It is important to note that these studies did not exclusively evaluate adherence to antihypertensive medications for PWH who had achieved viral suppression, an opportune time for targeted intervention for HIV associated comorbidities.

Adherence to antihypertensive medications is determined by many factors at the PWH and provider level, yet there is limited research that combines HIV providers and PWH perspectives to antihypertensive medication adherence to inform the development of interventions that would be acceptable by HIV providers and PWH.(8–12) PWH tend to prefer their HIV provider to take on the de facto primary care physician role, such as when it came to cardiovascular health but less so when it came to overall lifestyle and dietary improvements. (13–15) In contrast, physician surveys suggest that HIV providers prefer to focus on matters related to HIV and have variability in their comfort level and capacity to address non-AIDS related comorbidities(16, 17). For intervention development, the Capability, Opportunity, Motivation model of behavior (COM-B) provides a framework for defining barriers in behavioral terms and identifying specific behavioral targets to address nonadherence.(18) For example, Capability-related barriers encompass factors such as knowledge of hypertension risk, professional identity and comfort in HIV-related comorbities by HIV providers, while Opportunity-related barriers include medication side effects, effective communication of the benefits of antihypertensive medication adherence. Motivational barriers may involve beliefs regarding medication efficacy, such as the perceived need for continuous medication intake or pill burden and interpretation of symptoms.(18) This article combined HIV providers and PWH perspectives to inform the development of intervention strategies that would be acceptable to both groups. This study aimed to examine patient and provider perspectives on antihypertensive medication adherence among people with HIV using the COM-B framework to inform intervention development.

## METHODS

### Study Design and Participants

This was a qualitative study using semi-structured interviews with patients and providers. Separate interview guides were developed and piloted for PWH and HIV providers. PWH interview guide focused on barriers to medication adherence as it related to antihypertensive medications, such as personal experiences with medications, communication with their HIV providers and attributes of their hypertension medication they liked and disliked. The provider interview guide centered around provider perceived barriers to antihypertensive medication adherence among PWH under their care and their role in helping PWH manage their blood pressure and antihypertensive medication adherence. PWH were 18 or older with a confirmed HIV diagnosis, who at the time of interview had an undetectable viral load (< 200 copies/mL) within the past year, diagnosed with hypertension, and who were prescribed antihypertensive medications. HIV providers consisted of clinicians (i.e. infectious disease physicians, internists, or advance practice practitioners) with PWH under their care within the past 6 months.

### Data Collection

Interviews were conducted from August 2022 to February 2024. PWH participants were recruited from the Duke University Medical Center (DUMC), and HIV providers were recruited from different academic medical centers in south-eastern United States including DUMC using purposive sampling.(19) Semi-structured interview guides were developed for this study to explore patient and provider perspectives on antihypertensive medication adherence (see Supplementary File 1). The rationale for conducting the interviews of PWH at a single site was to generate in-depth, context-specific insights to inform the design of tailored intervention strategies, an approach shown to enhance intervention acceptability, feasibility, and sustainability within real-world clinical settings.(20, 21) This approach is consistent with implementation science frameworks emphasizing the critical role of local context in intervention design and uptake. Eligible PWH participants were identified through clinic records and approached by study staff either in person or via phone. HIV providers were identified through the clinic providers roster and a notification for study participation was also posted on the Center for AIDS Research website. After obtaining verbal consent from participants, interviews were led by a study team member (MB) who is trained in qualitative research, and the interviewer did not have prior relations with the participants. Interviews were conducted via phone or secure video platform (Zoom) depending on participant preferences and recorded with an encrypted digital recorder. At the beginning of each interview, participants were informed of the interviewer’s role as a member of the research team and the purpose of the study. Only one PWH opted out of audio recording, and for this one interview, extensive notes were taken throughout the interview. Debrief notes were taken for all interviews. Initial field notes were taken during and after interviews to document contextual observations and reflections using Dedoose. PWH interviews lasted no longer than 45 minutes. All provider interviews were approximately 30 minutes. PWH and Provider interviews were transcribed by an institution-approved transcription service. Transcripts were not returned to participants, and participants did not review findings. No non-participants were present during the interview, and no interviews were repeated.

Participants received a $50 incentive, with a few HIV providers opting out of receiving the incentive.

### Data Analysis

The study team used a rapid analysis approach with theme matrix techniques(22), involving a coding tree structure using Excel. Priority interview guide questions were chosen at both the PWH and provider level, and priority questions were organized into concepts. With these concepts, a matrix was developed, and individual responses from transcripts were summarized separately for each of these groups. After individual summaries for each participant were complete for both PWH and HIV providers, summaries were done across participants.

Authors JK and MB met together extensively to encode concepts and discuss patterns and themes around barriers and facilitators to antihypertensive medication adherence noticed throughout the initial analysis process, and such themes were further revised through collaborative team discussions with differing perspectives clarified with a group consensus. This work was conducted in line with the Consolidated Criteria for Reporting Qualitative Research (COREQ) checklist.

### Behavioral Framework

After analysis, themes identified were then described in terms of the domains of the Capability, Opportunity, Motivation Behavior (COM-B) model, a framework used to identify modifiable behaviors as targets for behavior change interventions. (18) Capability (C) refers to the individual's capacity to engage in a particular behavior which includes both physical and mental abilities, such as knowledge, skills, memory, attention, and physical strength. Opportunity (O) encompasses external factors facilitating or promoting the behavior that can arise from social cues, cultural influences, physical surroundings, or the availability of resources. Motivation (M) involves the mental processes driving behavior of why individuals choose to engage in a particular behavior.(18) We further identified the overlapping domains for PWH and HIV providers.

### Ethical approval

This study was approved by the Duke University Medical Center (DUMC) Institutional Review Board (IRB). All procedures involving human participants were performed in accordance with the Declaration of Helsinki.

## RESULTS

### Participants Characteristics

The sample consisted of 20 PWH, predominantly male (n=12, 60%) and Black/African American (n=15, 75%) with many aged 60–68 years (n=12, 60%). Most (n=17, 85%) had been living with HIV for over 10 years with chronic experiences of HIV.

The provider sample comprised 19 HIV healthcare providers with an even sex distribution (9 female, 9 male, 1 declined to answer). The majority of providers were White (n=12, 63%) and younger, as nearly half (n=9, 47%) were in the age range of 30–40 years. The vast majority of these providers (n=16, 84%) have provided services to 50+ individuals living with HIV yearly, with almost half (n=9, 47%) overseeing 100+ PWH, describing long-term experiences in HIV care. A summary of the demographic characteristics for both groups can be displayed in Tables 1 and 2.

### Perspectives of Antihypertensive Medication Adherence

Across interviews, PWH and HIV providers described a largely convergent set of barriers to antihypertensive medication adherence, though they differed in how these barriers were experienced, discussed, and addressed in clinical encounters. Shared themes included concerns about side effects, pill burden, competing life demands, limited salience of hypertension relative to HIV, and gaps in communication.

### Medication Side Effects

Both PWH and HIV providers identified actual or anticipated side effects as a major contributor to nonadherence. PWH described concerns informed by individual experiences, observing others, or self-directed research. Side effects described included frequent urination, headaches, fears of reduced fertility, and potential allergic reactions. These concerns often led to intentional discontinuation of antihypertensive medication taking, sometimes for prolonged periods. As one PWH recounted after finding out that their antihypertensive medication could potentially lower sperm count:

“…I did some research on it [bp med], and it said that it would affect my –– sperm count. I wouldn’t produce as much now as I did before… It said it would have a great effect on that. And, well, I—I didn’t wanna take it. But to be truthful with you, I did stop taking it[bp med] for three months.”

[Male, Age 63, More than 10 years HIV]

Another PWH admitted to sometimes not taking a couple of prescribed antihypertensive medications because it caused them to have to urinate often and at times wet themselves and drastically interfere with their day-to-day activities.

“I usually don’t take it [bp medications] because I have so much I have to do during the day…it’s kind of like sometimes, you’ve got to take the bitter with sweet. But sometimes, the bitter outweighs the sweet and you’re like, “Nuh-uh. I just can’t do it.” And I know it’s affecting my blood pressure and my heart and stuff, but I have to weigh it out.”

[Female, Age 58, More than 10 years with HIV]

HIV providers similarly recognized side effect concerns as a common reason PWH inconsistently took or stopped taking antihypertensive medications. While HIV providers viewed these concerns as modifiable through counseling or medication adjustment, PWH frequently weighed side effects against perceived benefits and concluded that the tradeoff was not worthwhile, particularly in the absence of symptoms.

“One issue that I’ve seen a fair bit, particularly with patients who have hypertension but not severe hypertension, is hesitancy around starting other medications…. And the idea of adding on something else people just have concerns, “Oh, am I gonna have side effects? How’s this going to interact with other medications?” And then I think hypertension in particular there’s this sort of unique challenge that blood pressure goes up and it comes down. I think there’s a lot of misperceptions around what that means. And, “Oh, well let’s just wait a little bit more and see if it gets better.”

[Male, Age 36, Provider treats more than 100 HIV patients each year]

### Pill Burden

Pill burden emerged as one of the most consistent areas of alignment between PWH and HIV providers. PWH described feeling overwhelmed by the cumulative number of medications required to manage HIV alongside other chronic conditions, expressing frustration with the prospect of adding another lifelong medication. For some, the addition of antihypertensive medications prompted intermittent nonadherence or complete discontinuation of medications. As one PWH stated:

“So, in the beginning when I first was diagnosed with HIV, I had four [pills]. And I did it, but it was overwhelming. So, then, when I got down to one, I was happy, but then, now all these other issues started, playing around with as far as the blood pressure. So, I honestly stopped taking everything for almost a year. I was like, forget it. I’m not taking any more medicine. I’m sick of it.”

[Female, Age 52, More than 10 years with HIV]

HIV providers echoed these observations, emphasizing that nonadherence was rarely due to disbelief in the hypertension diagnosis but rather to the cumulative burden of polypharmacy and the cognitive and logistical challenges of managing multiple daily medications. As one provider states about pill burden:

“I don’t really see in my patients going, ‘I don’t believe I have hypertension, I’m not gonna take this pill.’ It’s more of just the pill burden of, ‘I’m taking a pill every day already.’ And a lot of my patients are taking multiple pills, so it’s just kind of the poly pharmacy and the pill burden and keeping track of what goes where.”

[Male, Age 36, HIV provider who cares for 50–100 HIV PWH each year]

Both groups framed pill burden as a persistent challenge rather than a transient adjustment issue, positioning it as a shared opportunity for intervention.

### Competing Life Demands and Forgetfulness

PWH and HIV providers consistently described how busy, unpredictable schedules and competing responsibilities interfered with consistent medication use. PWH cited caregiving roles, demanding or irregular work schedules, recovery from acute health events, and general exhaustion as reasons for missed doses. Some PWH also described a more diffuse reluctance to take antihypertensive medications, particularly on days when hypertension felt abstract or inconsequential.

As one PWH described:

“I just didn’t feel like taking it….sometimes, I would miss it a day or two. And then, I would take one and do it for the rest – take it then – I may skip another day…to be honestly speaking with you, I don’t really like taking it, taking the blood pressure medicine. I feel like – they say I’m hypertension and stuff. But a lot of times when I go, everything seems to be okay. But I get this because I’m taking the medicine. And if I wasn’t taking the medicine, it probably would be different.”

[Male, Age 62, More than 10 years with HIV]

HIV providers similarly attributed missed doses to forgetfulness and competing priorities, agreeing that hypertension often falls lower on daily agendas for PWH compared with more immediate concerns.

“…they know that if they take and stop their HIV medication that they’ll build resistance. They’ve learned that and it’s ingrained in them. But if they forget their hypertension medication, and then they try to take it just before they come clinic to pass the test, it doesn’t always work. So, I think their perceived risk of not taking is not as high. I think the penalty’s not as – I think the perception is the penalty’s not as bad if they miss their hypertension medication. And what will kill them they think is HIV, so they’re gonna be very particular with that one. And the other one that we’ll try, and some people are good about it, but others forgive themselves for forgetting their hypertension meds.”

[Female, Age 44, Provider treats 50–100 HIV patients each year]

### Hypertension as a Lower-Priority Condition Compared With HIV

Both PWH and HIV providers emphasized the relative invisibility of hypertension compared with HIV. PWH frequently noted that they did not “feel” hypertension and questioned its seriousness when blood pressure readings appeared normal, reinforcing intermittent adherence. HIV providers reinforced this perspective, explaining that hypertension rarely provides immediate feedback analogous to HIV viral load measurements.

“I don’t look at it [high bp] as being serious. Maybe I should but I don’t. I guess, because there’s so many more other things going on that’s to me, it seems to be more important…I don’t feel that my high blood pressure is a top priority. But I take my medicine, but I don’t really be that concerned about it.

[Female, Age 60, More than 10 years with HIV]

HIV providers acknowledged that they had developed refined, effective communication strategies around HIV adherence but lacked equally compelling narratives for hypertension prevention. This gap limited their perceived capability to motivate PWH to prioritize antihypertensive adherence, despite recognizing its long-term importance.

“I think it's [hypertension] not top of mind for them at all. I don't think this is unique to people living with HIV. I just think it's very easy to kind of point to viral loads and draw diagrams. I think we have really good conversations around that. And maybe I need to do a better job of trying, you know. I've got kind of a spiel that I have for HIV, and adherence, and stuff that I've kind of honed over the years. I don't think I've been quite as intentional about thinking about, how do I sit down and communicate about hypertension to patients living with HIV? I mean, I do it, but – maybe it's not quite as polished.”

[Provider did not want to provide gender or age, HIV provider treats more than 100 HIV PWH each year]

This alignment highlights competing life demands as a shared barrier rooted in opportunity constraints rather than lack of awareness alone.

### Communication Gaps and Non-Disclosure of Nonadherence

A critical area of overlap involved recognition—implicit among PWH and explicit among HIV providers—that antihypertensive nonadherence often goes undisclosed. PWH described the lack of clear guidance from HIV providers on how to take them, especially when several different prescriptions were involved. They recounted misunderstandings about which pills should be taken together and at what times of the day, which led to mistakes or missed doses. For some, this lack of clarity made managing their antihypertensives feel overwhelming and contributed directly to their irregular use.

As one PWH described:

“…because there was a miscommunication between myself and the doctor, I was going through a period where I was changing medications, and he added one on. And I thought it was to replace a current medication that I was on. And I didn’t realize I was supposed to be taking it in addition to. And so I was only taking one of my two medications, and my numbers were running higher than normal. And he says, “You’re taking both of your meds, aren’t you?” And I’m, “What you mean I’m taking both of the meds? I’m taking one of them.” He’s, “Why?” So, I said, “Oh, I guess I misunderstood that when you gave me the new med, it was in addition to the first one. And I thought I was replacing one and not adding on.”

[Male, Age 59, 6–10 years with HIV]

PWH described intentionally withholding information about missed or stopped medications due to fear of judgment, discomfort discussing sensitive issues, or confidence that nonadherence would go unnoticed. Unlike HIV care, where missed doses are quickly revealed through laboratory monitoring, PWH perceived blood pressure control as more ambiguous and therefore easier to conceal.

“Well, I didn’t feel comfortable talking with her about it because of the – not be able to produce more sperm than I would have. And that’s because she’s a female…. I felt as if she would tell me that I had to stay on that same medication, that she couldn’t change it. And I suppose I shoulda went on and talked to her about it, and she probably would’ve been able to change it. But I don’t know. I just – I concentrated more on the negative than I should have on the positive.”

[Male, Age 63, HIV more than 10 years]

HIV providers indirectly corroborated this dynamic by describing variability in blood pressure readings and difficulty distinguishing transient elevations from sustained poor control.

As one provider said:

“I think people feel maybe that they've got more agency over their own cholesterol control, because they know what they do every single day, from a dietary and exercise point of view. Whereas, that link of, ‘Hey, this is within my ability to control it,’ I don't think they feel as strongly for hypertension. And so, I get many more people coming in talking to me concerned about their cholesterol values than I ever do about blood pressure.”

[Male, Age 46, HIV provider treats 20–49 PWH each year].

This shared context contributed to missed opportunities for timely intervention and reinforced nonadherence as a socially mediated barrier within the **opportunity** domain of COM-B.

### Provider Capacity and Role Constraints in Hypertension Management

HIV providers uniquely emphasized structural and professional constraints that shaped their ability to address antihypertensive adherence, though these constraints had downstream implications for PWH. Infectious disease specialists described frequently assuming primary care responsibilities for PWH without dedicated primary care physicians, particularly for hypertension management.

As one provider explains:

“In a perfect world, I think I would love to be the person who helps manage all of those conditions for my HIV-infected PW. In the imperfect world, I don't know that I would necessarily have the time for all of that because our well-controlled HIV infected PWH, we see them at this point every six months, and I think if you are managing those conditions more aggressively, things like hypertension, diabetes, cholesterol, you will need to see them more frequently. And it's helpful I think to have not just the provider but kinda the resources in the clinic that are geared towards helping manage other chronic conditions as well, and I don't think our clinic necessarily has that right now.”

[Female, Age 40, HIV provider treats less than 20 HIV PWH each year]

While many felt comfortable managing uncomplicated hypertension, they expressed concern about limited clinic time, infrequent follow-up, lack of ongoing education in hypertension guidelines, and uncertainty around escalating therapy.

As one provider stated:

“I think that a lot of us are better managing the HIV than we are of the other things, such as high blood pressure or diabetes. And so, I think that it’s easy to have a patient encounter where the whole discussion ends up being about how great it is that the patient’s HIV’s controlled and forget to ask like, “Oh, how are you doing with your blood pressure medications?” Particularly if, on that day, the patient’s blood pressure isn’t elevated.”

[Female, Age 37, HIV provider treats 50–100 PWH each year]

And another one stated:

“…. the other thing too that makes me anxious is I’m not doing any CME, continuing medical education, and blood pressure medications. I know the general stuff, like what goal BP is and what the most entry level blood pressure medicines are, but sometimes it seems like there's been changes or new meds out there; I’m not getting any kinda CME on that.”

[Female, Age 54, HIV provider treats more than 100 HIV PWH each year].

Table 3 summarizes key barriers and facilitators identified by patients and providers, mapped to COM-B domains.

## DISCUSSION

Despite advances in antiretroviral therapy and sustained viral suppression, PWH continue to experience a disproportionate burden of cardiovascular disease, with hypertension remaining a key contributor. (1, 2, 15) In this study, PWH and HIV providers identified largely overlapping barriers to antihypertensive medication adherence, though they differed in how these barriers were experienced and addressed. These findings highlight actionable opportunities to strengthen hypertension management within HIV care by addressing motivation, structural constraints, and communication. These findings can be understood through the COM-B framework, which highlights how interacting behavioral domains shape antihypertensive medication adherence. (18)

While prior studies have identified barriers such as pill burden, side effects, and competing priorities among PWH, this study advances the field by integrating both patient and provider perspectives within a virally suppressed population; a critical but underexamined stage of care. Rather than identifying new barriers, our findings clarify how these barriers persist and interact in the context of successful HIV treatment, where clinical attention shifts toward long-term comorbidity management. Importantly, these findings demonstrate how established HIV care structures, particularly adherence monitoring, communication strategies, and longitudinal patient-provider relationships, can be leveraged to address antihypertensive medication adherence.

Approaches such as motivational interviewing, patient-centered education, and regular shared decision-making with prescribers may help sustain adherence by positioning patients as active participants in their care and aligning treatment plans with their priorities and lived experiences. (23) Motivational interviewing, in particular, has been associated with improved medication adherence and clinically meaningful reductions in blood pressure in prior studies, proposing potential to address ambivalence toward long-term treatment.(24) Similarly, shared decision-making frameworks have been shown to improve treatment engagement and adherence in chronic disease management by fostering patient autonomy and trust.(25, 26) In addition, simplifying treatment through fixed-dose combination antihypertensive therapy may reduce perceived burden and improve adherence, with evidence demonstrating modest but consistent improvements in adherence and reductions in adverse cardiovascular outcomes compared with multi-pill regimens.(23, 27, 28)

Structural and contextual barriers further constrained adherence. Both PWH and providers described how competing life demands, such as work, caregiving, and fatigue, disrupted medication routines. Missed doses often reflected breakdowns in daily structure rather than intentional nonadherence. These findings support evidence that stable routines and environmental cues are critical for adherence.(26) It may also be that these patterns reflect underlying difficulties with executive functioning (EF), a set of skills involved in planning, organization, emotional regulation, impulse control, and adjusting to unexpected changes.(29, 30) For PWH, managing complex treatment routines alongside other life demands may be especially difficult.(8, 31) Because EF challenges are frequently observed among PWH, (32–34) future research would benefit from including standardized EF assessments.

Unlike HIV care, where viral load monitoring provides clear feedback, blood pressure variability reduced the visibility of nonadherence and limited HIV providers’ ability to detect and respond to missed doses. This uncertainty, combined with competing clinical priorities, often shifted attention toward HIV management. Limited consultation time, infrequent follow-up for clinically stable patients, and fragmented care across multiple HIV providers reduced opportunities to address antihypertensive adherence proactively. At the same time, many patients relied on their HIV providers for broader chronic disease management, showing misalignment between patient expectations and care organization. (35, 36)

Communication dynamics further influenced adherence. PWH reported misunderstandings regarding medication instructions, particularly during regimen changes, and described withholding nonadherence due to fear of judgment or the perception that it would go unnoticed. Providers, however, did not consistently recognize these gaps. This disconnect highlights the need for clear, structured, and nonjudgmental communication approaches that normalize discussions of nonadherence and improve patient understanding. (23)

Strategies that strengthen habit formation and reduce contextual burden, such as medication reminders, synchronized refills, and community-based delivery systems, have demonstrated effectiveness in improving adherence by minimizing logistical barriers and supporting consistent routines.(23, 37) In parallel, more deliberate integration of hypertension management into HIV care workflows may enhance continuity and increase opportunities for adherence assessment and support, particularly where patients rely on a single provider for multiple chronic conditions. (38)

Capability-related barriers to antihypertensive medication adherence were evident across both patients and HIV providers, particularly in relation to risk communication, understanding of long-term consequences, and clinical preparedness to manage hypertension within HIV care. Both groups described difficulty translating hypertension into a meaningful and actionable condition. While patients were aware of their diagnosis, many struggled to understand long-term cardiovascular risk in the absence of symptoms. HIV providers similarly acknowledged having fewer refined tools and narratives to communicate hypertension compared with HIV, where adherence is supported by clear biomarkers and well-established messaging.(35, 36, 39) This gap in communication capability limited the effectiveness of adherence discussions and reduced patients’ ability to internalize the importance of sustained blood pressure control. (38)

Communication challenges were further reflected in misunderstandings around medication instructions, particularly during regimen changes or in the context of multiple prescriptions. Patients described confusion about dosing and medication combinations, which contributed to unintentional nonadherence. HIV providers, however, did not consistently recognize these communication gaps, suggesting a disconnect between information delivery and patient understanding. This aligns with evidence that limitations in health literacy, clarity of communication, and regimen complexity can undermine adherence, particularly in chronic disease settings requiring long-term self-management.

Provider-level constraints observed in this study included variability in confidence and limited access to structured guidance. These gaps are especially consequential where HIV providers serve as the primary point of care for multiple chronic conditions without parallel support systems.(40, 41)

Addressing these limitations will require strengthening both patient-facing communication and provider-facing clinical support. Developing clear, standardized communication approaches for antihypertensive medication adherence for PWH, analogous to the structured narratives used in HIV care, may improve patient understanding by translating long-term cardiovascular risk into more actionable and relatable concepts.(40, 41) In parallel, targeted provider training, simplified clinical algorithms, and decision-support tools may enhance consistency in hypertension management and reduce reliance on individual experience.

These findings have several implications for practice. Improving antihypertensive adherence in HIV care will require a more integrated approach that addresses patient motivation, structural barriers, and provider capability simultaneously. Simplifying treatment regimens, embedding routine adherence discussions, and using shared decision-making to align treatment with patient priorities may improve engagement. Strengthening provider support through communication tools, decision aids, and integration of hypertension management into HIV workflows may enhance consistency in care delivery. These domains are interdependent: when hypertension is poorly understood, it is deprioritized; when routines are disrupted, adherence becomes inconsistent; and when communication is limited, opportunities for intervention are missed. Leveraging the strengths of HIV care, structured adherence support, routine monitoring, and longitudinal relationships, offers a high-yield and scalable strategy to improve cardiovascular outcomes.

A limitation of this study is that data were collected within a single clinical setting, which may limit generalizability. However, this context-specific approach enabled the identification of actionable barriers directly relevant to intervention design.(20, 21) Future research should evaluate these findings across diverse settings and test implementation strategies that integrate hypertension management into HIV care delivery.(42) (43)

## CONCLUSION

Antihypertensive medication nonadherence among PWH reflects interconnected patient-, provider-, and system-level challenges not fully addressed within current HIV care models. Extending the strengths of HIV care to hypertension management offers a practical and scalable pathway to improving long-term cardiovascular outcomes in this population.

## Supplementary Material

This is a list of supplementary files associated with this preprint. Click to download.

- PatientIDIGuideSupplementaryFile1.docx

## Figures and Tables

**Table 1.** PWH Demographics

	N=20
**Sex**	
Female	8
Male	12
**Race**	
African American/Black	15
White	5
**Age**	
51–59	6
60–68	12
69–78	2
**Years since diagnosed with HIV**	
6–10 years	3
More than 10 years	17
	

**Table 2.** Provider Demographics

	N=19
**Sex**	
Female	9
Male	9
Refused	1
**Age**	
30–40	9
41–50	6
51–60	3
Refused	1
**Race**	
White	12
African American/Black	4
Asian	1
Other	1
Refused	1
**Number of PWH care for each year**	
Less than 20	1
20–49	2
50–100	7
More than 100	9

**Table 3.** Synthesis of Barriers and Facilitators Mapped to COM-B Domains

Theme	PWH	HIV providers	Overlap	COM-B Domain
Side effects	Experienced or anticipated side effects reduced willingness to take medications.	Recognized side effects as a common reason for nonadherence.	Side effects are widely acknowledged as a key adherence barrier.	**Motivation**
Pill burden	Felt overwhelmed by adding antihypertensives to lifelong HIV treatment.	Identified polypharmacy as a central driver of nonadherence.	Strong alignment around the pill burden.	**Motivation**
Low perceived urgency	Deprioritized hypertension due to lack of symptoms or normal readings.	Noted difficulty motivating adherence without immediate feedback.	Hypertension is viewed as less salient than HIV.	**Motivation**
Competing life demands	Busy schedules, caregiving, illness, and fatigue interfered with routines.	Attributed missed doses to competing priorities and forgetfulness.	Shared recognition of opportunity constraints.	**Opportunity**
Non-disclosure of nonadherence	Withheld missed doses due to fear of judgment or belief it would go unnoticed.	Described difficulty detecting nonadherence due to BP variability.	Antihypertensive nonadherence is perceived as less visible.	**Opportunity**
Medication taking communication	Misunderstood dosing or addition of medications.	Rarely identified miscommunication as a barrier.	Partial overlap; more salient for PWH	**Opportunity**
Understanding hypertension risk	Had difficulty appreciating long-term risk without symptoms.	Reported limited tools to communicate prevention effectively.	Shared challenge communicating hypertension risk.	**Capability**
Provider role & care integration	Relied on HIV providers for hypertension management.	Reported role strain but greater ease in integrated settings.	Integrated care and clear communication viewed as facilitators.	**Capability**

## Data Availability

The authors confirm that the data supporting the findings of this study are available in the article and its Supplementary Materials upon reasonable request. Raw data can be also made available upon reasonable request from the Corresponding Author.

## Abbreviations

PWH: People with HIV
HIV: Human Immunodeficiency Virus
CVD: Cardiovascular Disease
ART: Antiretroviral Therapy
COM-B: Capability, Opportunity, Motivation–Behavior
IRB: Institutional Review Board
DUMC: Duke University Medical Center
EF: Executive Function
